# Supplementary material for: Longevity interventions modulate mechanotransduction and extracellular matrix homeostasis in C. elegans
Source: Nat Commun. 2024 Jan 4;15:276. doi: 10.1038/s41467-023-44409-2 (PMC10766642; doi:10.1038/s41467-023-44409-2)
Supplement: Supplementary file 1 — Supplementary Information [file 41467_2023_44409_MOESM1_ESM.pdf]

SUPPLEMENTARY INFORMATION

Longevity interventions modulate mechanotransduction and extracellular matrix homeostasis in *C. elegans*

Alina C. Teuscher, Cyril Statzer, Anita Goyala, Seraina A. Domenig, Ingmar Schoen, Max Hess, Alexander M. Hofer, Andrea Fossati, Viola Vogel, Orcun Goksel, Ruedi Aebersold, Collin Y. Ewald

SUPPLEMENTARY FIGURES

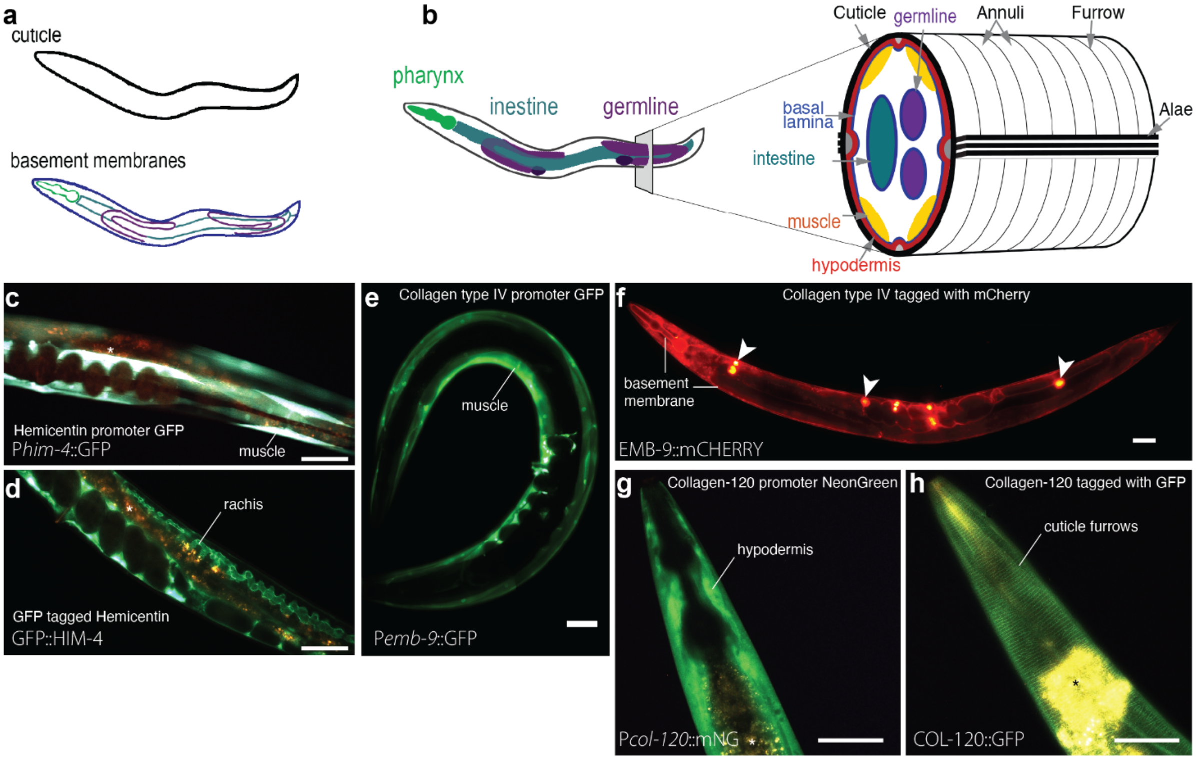

i Transcriptional Reporter

	Matrisome	Genotype	egg	L1-L3						L4						Day 1 adult						Day 8 adult					
			total	H	I	G	B	P	N	H	I	G	B	P	N	H	I	G	B	P	N	H	I	G	B	P	N
Core Matrisome	Cuticular Collagen	*Pcol-10* ::GFP	2	3						2						3						1	1				
Core Matrisome	Cuticular Collagen	*Pcol-19* ::GFP														2											
Core Matrisome	Cuticular Collagen	*Pcol-120* ::NeonGreen								3						2						1					
Core Matrisome	Cuticular Collagen	*Pcol-129* ::GFP	0.5	1	1					1	1								3				1				
Core Matrisome	Cuticular Collagen	*Pcol-144* ::GFP	1.5	2						3						3						1					
Core Matrisome	Cuticular Collagen	*Pcol-89* ::GFP							3		1	3p			3		2	2p			3		2	2p			3
Core Matrisome	Cuticular Collagen	*Pdpy-14* ::GFP	3	2						1						1	1					1	1				
Core Matrisome	Collagens	*Pemb-9* ::GFP	2				2			1			2						2						2		
Core Matrisome	Collagens	*Plet-2* ::GFP	2				2						2						2						1		
Core Matrisome	Collagens	*Pmec-5* ::GFP	2				2						3						2						2		
Core Matrisome	Cuticlin	*Pcut-6* ::GFP	1	1						1						1						3					
Core Matrisome	Cuticlin	*Pcutl-23* ::GFP	1	1	1				2		1	2			1	1		3p			3		1	1			
Core Matrisome	ECM Glycoprotein	*Phim-4*:: GFP					2						2				1		3				1		3		
Core Matrisome	ECM Glycoprotein	*Phim-4* ::MB::YFP	2				2						2						2						2		
associated	ECM Regulators	*Pcri-2* ::GFP	2	1	1		2			2	1		2			2			2			2			2		
associated	ECM-affiliated	*Pgpn-1* ::GFP			3			3			3			3		1	3			3			3				

fluorescent scale: 0, 0.5, 1, 1.5, 2, 2.5, 3

#p partially

C Cuticle
BM Basement Membrane
H Hypodermis
I Intestine
G Germine
B Bodywall Muscle
P Pharynx
N Neurons

j Translation Reporter

	Matrisome	Genotype	egg	L1-L3								L4								Day 1 adult								Day 8 adult							
			total	C	BM	H	I	G	B	P	N	C	BM	H	I	G	B	P	N	C	BM	H	I	G	B	P	N	C	BM	H	I	G	B	P	N
Core Matrisome	Cuticular Collagen	BLI-1::GFP										2								2								1							
Core Matrisome	Cuticular Collagen	COL-120::GFP												1						2		1						1							
Core Matrisome	Cuticular Collagen	COL-19::GFP																		2								3							
Core Matrisome	Cuticular Collagen	LON-3::GFP		1		1						1		2						2						2								1	
Core Matrisome	Cuticular Collagen	ROL-6::GFP		1		1	1					2		1	1					2		1	1					1							
Core Matrisome	Cuticular Collagen	COL-101::GFP										1								2								1							
Core Matrisome	Collagens	COL-99::GFP					1																												
Core Matrisome	Collagens	GFP::COL-99	3			3					2			3					2			1			1		2			1					1
Core Matrisome	Collagens	EMB-9::DENDRA	0.5		2	1				2			1	2	1	1		2			2	1	2	2		2			3	1	3	3		3	
Core Matrisome	Collagens	EMB-9::mCherry	3		1	1				2			2	1	2	2		2			2	1	2	2		2			3	1	3	3		3	
Core Matrisome	Cuticlin	CUTI-1::GFP	1.5			1								1								1	1							2	2				
Core Matrisome	ECM Glycoprotein	GFP::HIM-4	1.5		1			2		2	2		2	1		2		3	2		3	1		3		2	2		3			3		1	
Core Matrisome	ECM Glycoprotein	LAM-1::DENDRA	1		1	1					2		1	1					2		2	1					2		2	2					3
Core Matrisome	ECM Glycoprotein	LAM-1::mCherry				1							1	1							2	1	2	2		2			3	1	3	3		3	
Core Matrisome	ECM Glycoprotein	LAM-2::mKate2 *	1		1	1							2	1							2	1		2		1	2		2	1		2		1	2
Core Matrisome	Proteoglycans	mNG::UNC-52 *	0.5		1								1								2			2					3			3			
associated	ECM-affiliated	SDN-1::GFP	3			1	1				2			1	1				2			1					1			1					3
Adhesome	Integrin	INA-1::GFP	2			1	1			1	2			1	1	3		1	1			1	2	2		2					2	3		2	
Adhesome	Integrin	PAT-2::mNG *	2						1								2							1	2							1	2		
Adhesome	Integrin	PAT-3::GFP *	3						3							2	3					1		2	3							1	3		
Adhesome	Fibronectin-like	LET-805::GFP *	2		2				2	2			2			1	2	2			2			1	2	2			3			1	2	2	
Adhesome	Plectin	VAB-10a::GFP *	1.5				2							2								2								3					
Adhesome	Talin	GFP::TLN-1 *	1.5			1			2					1			2					1		1	3					1			1		

Supplementary Fig. 1. Age-associated changes of matrisome proteins in the ECM
(a, b) Schematic of *C. elegans'* tissues and ECMs (basement membrane and cuticle).
(c, d) Expression of hemicentin *him-4* promoter-driven in body wall muscles (c) and GFP tagged HIM-4 protein incorporated in ECM (d).
(e, f) Expression of collagen type IV *emb-9* promoter-driven in body wall muscles (e) and mCherry tagged EMB-9 protein incorporated in almost all basement membranes surrounding pharynx, intestine, gonad, and apical surface of body wall muscles (f). Arrowheads indicate coelomocytes, the *C. elegans'* macrophage-like cells, which scavenge foreign particles, including fluorescent proteins from the pseudocoelomic fluid.
(g, h) Expression of cuticular collagen *col-120* promoter-driven in the hypodermis (g) and GFP tagged COL-120 protein incorporated in the cuticular furrows (h).
(i) Transcriptional reporters are driven by matrisome genes during development and aging. For details, see Supplementary Table 1. The fluorescent scale corresponds to the highest observed fluorescence of a reporter line (intensity 3) graded to no observed fluorescence above the background (intensity 0).
(j) Translational reporters of matrisome and adhesome proteins are localized and incorporated into ECM structures during development and aging. For details, see Supplementary Table 1. * indicates CRISPR-Cas9 genome inserted tag in the endogenous gene locus.
(c-h) scale bar = 50 µm, (c,d,g,h) * autofluorescent gut granules are in brown-yellowish.

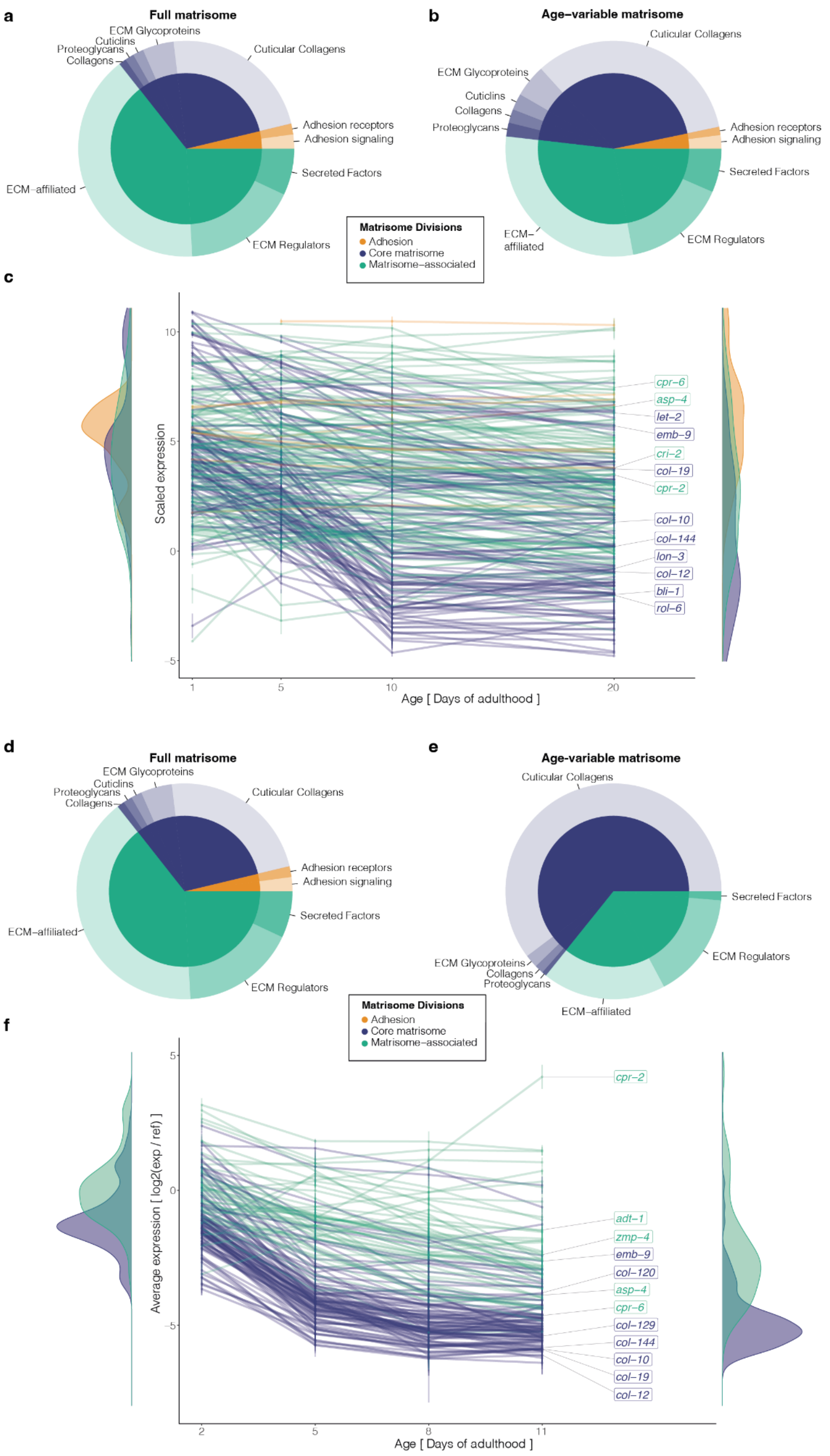

a
Full matrisome
ECM Glycoproteins
Cuticlins
Proteoglycans
Collagens
Cuticular Collagens
Adhesion receptors
Adhesion signaling
Secreted Factors
ECM–affiliated
ECM Regulators
b
Age–variable matrisome
Cuticular Collagens
ECM Glycoproteins
Cuticlins
Collagens
Proteoglycans
Adhesion receptors
Adhesion signaling
Secreted Factors
ECM–affiliated
ECM Regulators
Matrisome Divisions
Adhesion
Core matrisome
Matrisome–associated
c
Scaled expression
Age [Days of adulthood]
cpr–6
asp–4
let–2
emb–9
cri–2
col–19
cpr–2
col–10
col–144
lon–3
col–12
bli–1
rol–6
d
Full matrisome
ECM Glycoproteins
Cuticlins
Proteoglycans
Collagens
Cuticular Collagens
Adhesion receptors
Adhesion signaling
Secreted Factors
ECM–affiliated
ECM Regulators
e
Age–variable matrisome
Cuticular Collagens
Secreted Factors
ECM Regulators
ECM Glycoproteins
Collagens
Proteoglycans
ECM–affiliated
Matrisome Divisions
Adhesion
Core matrisome
Matrisome–associated
f
Average expression [log2(exp / ref)]
Age [Days of adulthood]
cpr–2
adt–1
zmp–4
emb–9
col–120
asp–4
cpr–6
col–129
col–144
col–10
col–19
col–12

Supplementary Fig. 2. Transcriptional changes of matrisome and adhesome during aging

(a, b) Composition of the matrisome and adhesome (a) and their age-variable subset (b) based on the GSE12168 expression profile (b).

(c) Longitudinal expression of matrisome and adhesome genes during aging (GSE12168).

(d, e) Composition of the matrisome and adhesome (d) and their age-variable subset (e) based on the GSE46051 expression profile.

(f) Longitudinal expression of matrisome and adhesome genes during aging (GSE46051).

(a-f) For details, see Supplementary Table 2.

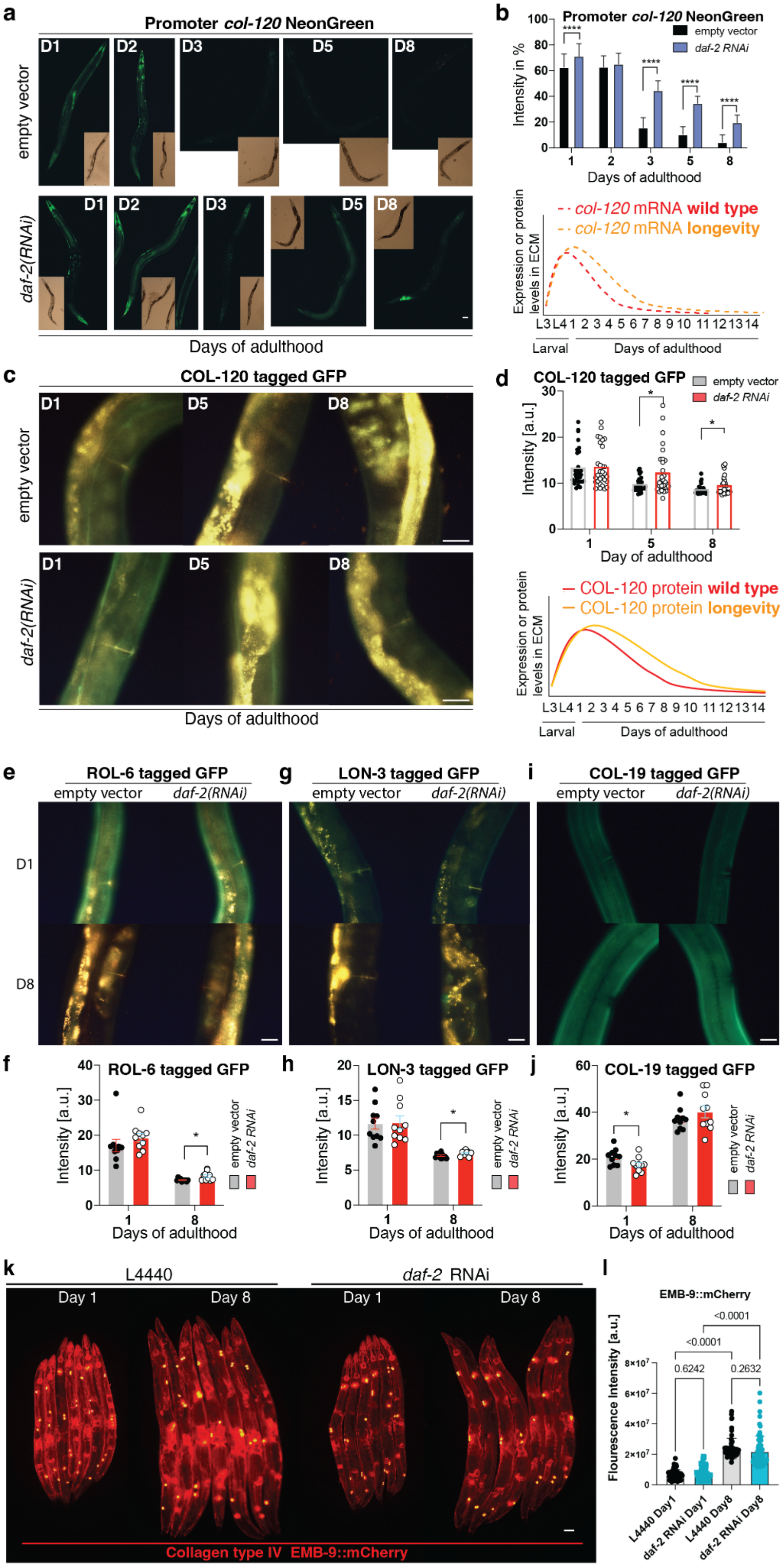
a
Promoter col-120 NeonGreen
empty vector
daf-2(RNAi)
D1
D2
D3
D5
D8
Days of adulthood
b
Promoter col-120 NeonGreen
empty vector
daf-2 RNAi
Intensity in %
Days of adulthood
col-120 mRNA wild type
col-120 mRNA longevity
Expression or protein levels in ECM
Larval
Days of adulthood
c
COL-120 tagged GFP
d
COL-120 tagged GFP
Intensity [a.u.]
Day of adulthood
COL-120 protein wild type
COL-120 protein longevity
e
ROL-6 tagged GFP
g
LON-3 tagged GFP
i
COL-19 tagged GFP
f
h
j
Days of adulthood
k
L4440
daf-2 RNAi
Day 1
Day 8
Collagen type IV EMB-9::mCherry
l
EMB-9::mCherry
Flourescence Intensity [a.u.]
L4440 Day1
daf-2 RNAi Day1
L4440 Day8
daf-2 RNAi Day8

Supplementary Fig. 3. Collagen levels in the ECM during aging and longevity

(a) Time course of LSD1107 *Pcol-120*::NeonGreen animals fed with *daf-2* RNAi or the empty RNAi vector control L4440 bacteria. Scale bar = 50 µm.

(b) Quantification and model of the LSD1107 P*col-120*::NeonGreen time course. Three rounds, each n=20, were quantified using a visual grading scale system with values from 0 - 3 in 0.5 steps. Error bars represent SDs. For details and data, see Supplementary Table 4.

(c) Images of LSD2043 COL-120::GFP animals fed with *daf-2* RNAi or the empty RNAi vector control L4440 bacteria during aging, taken on day 1, day 5, and day 8 of adulthood. Scale bar = 25 µm

(d) Quantification of LSD2043 COL-120::GFP green fluorescence intensity levels shown as a composite of 3 independent biological trials of each about 10 *C. elegans* per condition and day. For details and data, see Supplementary Table 4. Below is the model of COL-120 levels in ECM during aging and upon longevity.

(e, g, i) Images of LSD2022 ROL-6::GFP, LSD2063 LON-3::GFP, and LSD2064 COL-19::GFP animals fed with *daf-2* RNAi or the empty RNAi vector control L4440 bacteria during aging, taken on day 1 and day 8 of adulthood. Scale bar = 25 µm.

(f, h, j) Green fluorescence intensity quantification of two separate experiments, each three rounds of *daf-2* or L4440 RNAi experiments on translational cuticular collagen reporter strains. The green fluorescence intensities (excluding autofluorescence from gut granules) of images of either 3 independent trials of about 10 animals (LON-3, COL-19) or 1 trial of 10 (ROL-6) animals were quantified (see Materials and Methods for details). The data are represented as mean and SD. * indicates *P*-value ≤ 0.05 determined by using a two-way ANOVA. For details and data, see Supplementary Table 4.

(k - l) An orthologue of mammalian Type IV collagen EMB-9 increases with age independent of slowing aging upon reduced Insulin/IGF-1 signaling. (k) Representative images of EMB::mCherry animals treated from eggs with empty vector (L4440) or *daf-2*(RNAi) and scored at day 1 and day 8 of adulthood at 20°C. Scale bar = 50 µm. (l) Quantification of EMB-9::mCherry fluorescent intensity. Each dot represents an animal. 3 independent biological trials. *P*-value determined with One-way ANOVA. For raw data and statistics, see Supplementary Table 4.

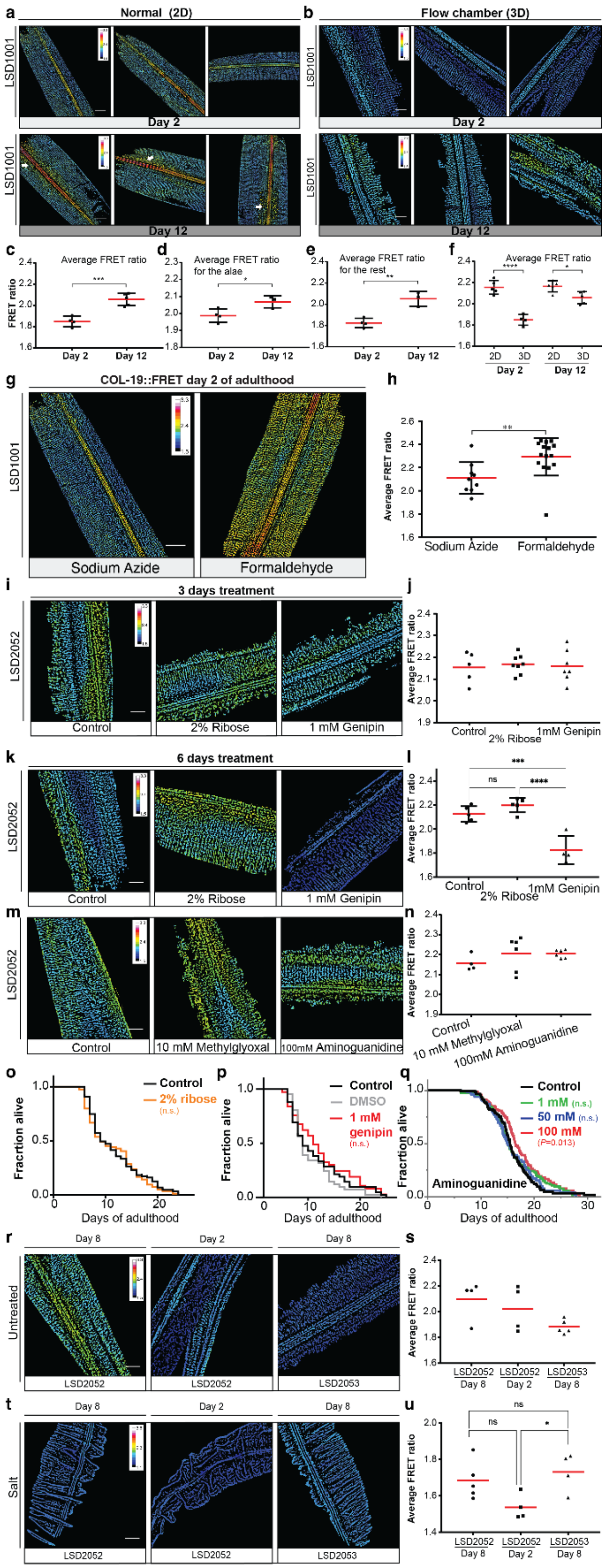
Normal (2D)
Flow chamber (3D)
LSD1001
Day 2
Day 12
Average FRET ratio
Average FRET ratio for the alae
Average FRET ratio for the rest
FRET ratio
COL-19::FRET day 2 of adulthood
Sodium Azide
Formaldehyde
Average FRET ratio
3 days treatment
6 days treatment
LSD2052
Control
2% Ribose
1 mM Genipin
10 mM Methylglyoxal
100mM Aminoguanidine
Fraction alive
Days of adulthood
2% ribose
DMSO
1 mM genipin
Aminoguanidine
1 mM
50 mM
100 mM
Untreated
Salt
LSD2053
Day 8

Supplementary Fig. 4. Collagen FRET-reporter implicates tissue tension and crosslinking associated with aging

(a-f) Comparison of FRET ratio images of transgenic LSD1001 COL-19::FRET animals imaged using normal (2D) or our developed flow chambers (3D) on day 2 and day 12 of adulthood and corresponding quantification.
(g) Representative FRET ratio images of transgenic LSD1001 COL-19::FRET animals anesthetized with 25 mM sodium azide or fixed with 4% formaldehyde. Scale bar = 20 µm, FRET calibration bar between 1.5 - 3.3,
(h) Quantitative analysis of FRET ratios from the whole cuticle revealed significantly higher FRET ratios for LSD1001 COL-19::FRET *C. elegans* fixed with 4% formaldehyde for 24 hours compared to LSD1001 COL::FRET *C.elegans* than were anesthetized with 25 mM sodium azide.
(i-n) Representative FRET ratio images and quantification of transgenic LSD2052 COL-19::FRET animals treated from day 1 of adulthood with different chemicals and scored at day 4 (i, j) or day 7 of adulthood (k-n).
(o-q) The lifespans of *C. elegans* were treated with chemicals starting during adulthood (Raw data and statistical details in Supplementary Table 7).
(r-u) Lowering inner pressure by osmotic conditions. On day 2 or 8 of adulthood, either normal-lived (LSD2052) or longevity-promoting (LSD2053) transgenic COL-19::FRET animals were picked and placed directly either into a physiological buffer or high sodium chloride (1g NaCl/ 10mL M9 buffer) containing flow chambers and then imaged.

(c-f, h, j, l, n, s, u) Error bars correspond to the standard deviation of the mean, * $P < 0.05$, ** $P < 0.01$, *** $P < 0.001$, **** $P < 0.0001$. Statistically significant differences between mean values were calculated using an unpaired *t*-test. For raw data, details, and statistics, see Supplementary Table 6.

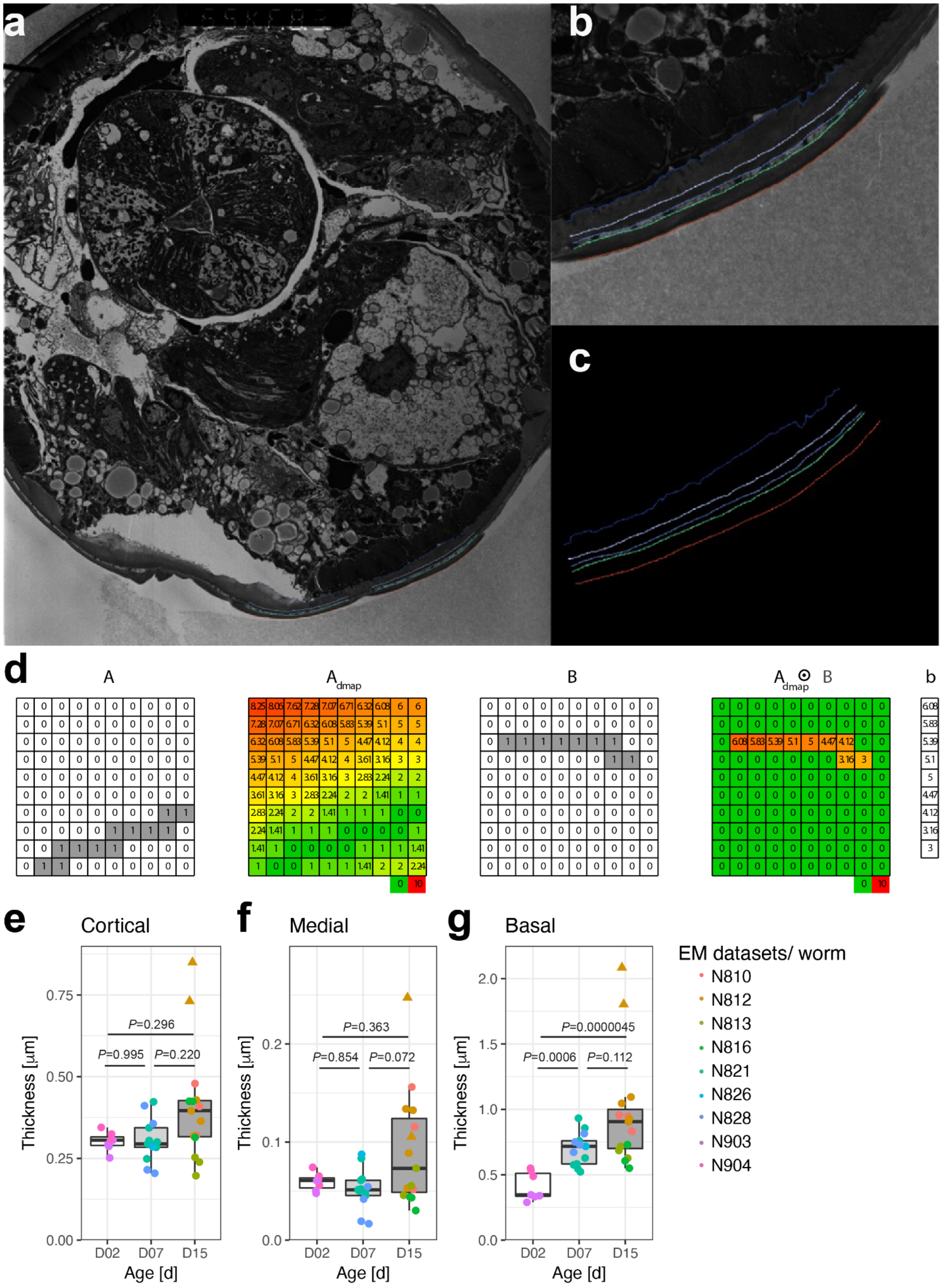

Supplementary Fig. 5. Cuticle thickness increases during aging
(a) Transverse transmission electron micrograph of a 15-day-old adult (Source: wormimage.org). Colored curves were drawn manually in regions included in the analysis of cuticle thickness.

(b) Zoom into a region used for evaluation.
(c) Colored curves correspond to binary masks exported to Matlab for evaluation.
(d) Workflow to measure the thickness of annotated cuticle layers. A = Binary mask of first layer border. A_{dmap} = Distance transform of A. B = Binary mask second layer border. $A_{dmap} \odot B$ = Elementwise multiplication of A_{dmap} and B, non-zero values correspond to the shortest distances to A for every pixel in B.
(e-g) Quantification of the thickness of (e) cortical (f) medial and g) basal cuticle. Dots correspond to individual animals. Triangles indicate outliers. Boxplot shows the median (black line), 25th/75th percentiles (hinges), and 1.5*IQR (whiskers). *P*-values are One-way ANOVA post hoc Tuckey without including outliers. See Fig. 3e for total cuticle thickness and Supplementary Table 9 for details.

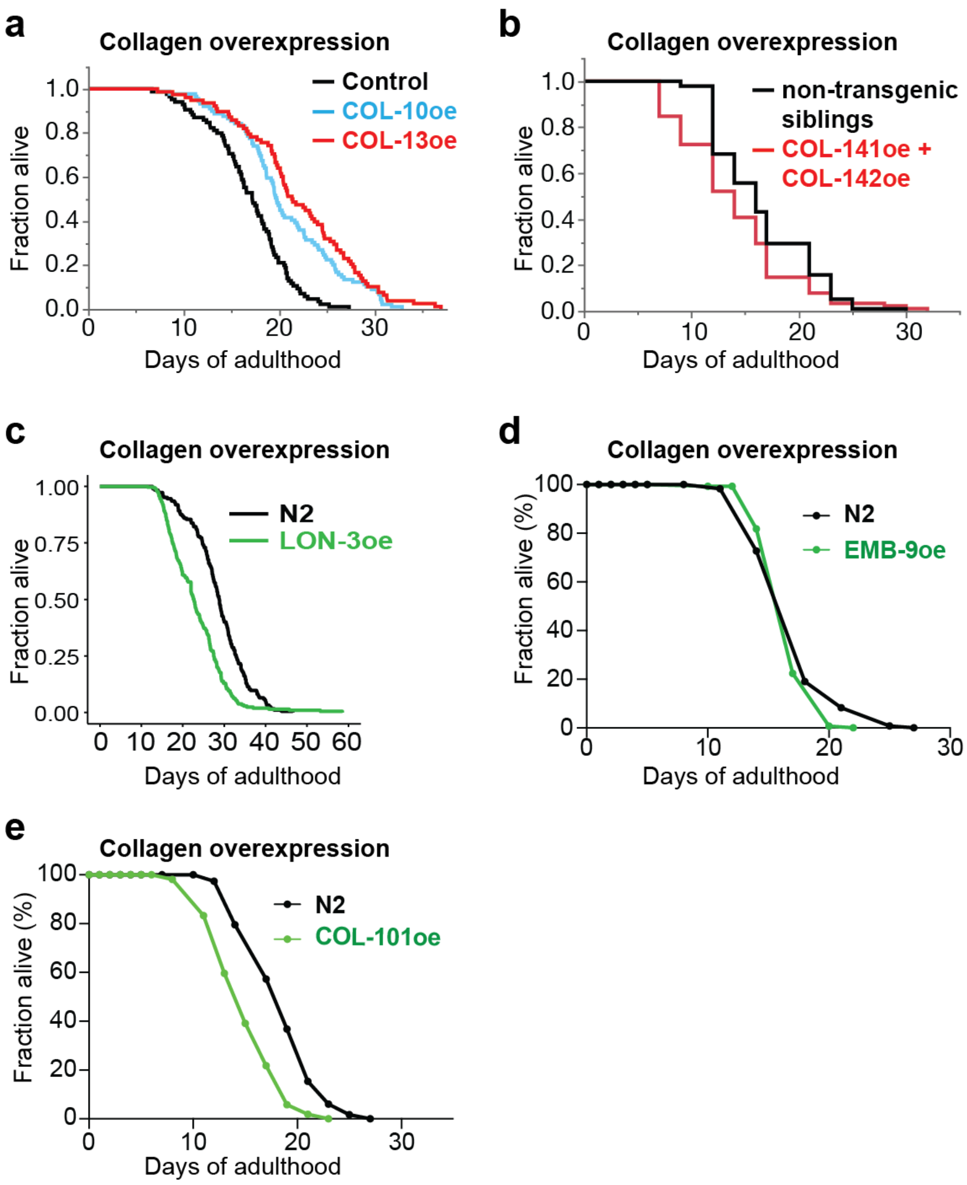

Supplementary Fig. 6. Only key collagen overexpression is sufficient to increase the lifespan

(a) Cuticular collagens COL-10 (LSD2018) or COL-13 (LSD2014) overexpression (oe) increased lifespan compared to control (wild type with *rol-6(su1006)* co-injection marker LSD2013) on UV-inactivated bacteria.

(b) Cuticular collagens COL-141+COL-142 (CS637) overexpression (oe) did not extend lifespan compared to non-transgenic sibling control.

(c) Cuticular collagen LON-3 (*kuIs55*) overexpression (oe; 8x outcrossed to N2) did not extend lifespan compared to wild type (N2).

(d) Basement membrane Type IV collagen EMB-9 (NK364) overexpression (oe) did not increase lifespan compared to wild type (N2).

(e) Cuticular collagen COL-101 (*dmaIs40*) overexpression (oe) shortened lifespan compared to wild type (N2).

(a-e) For details, raw data, and statistics, see Supplementary Table 7.

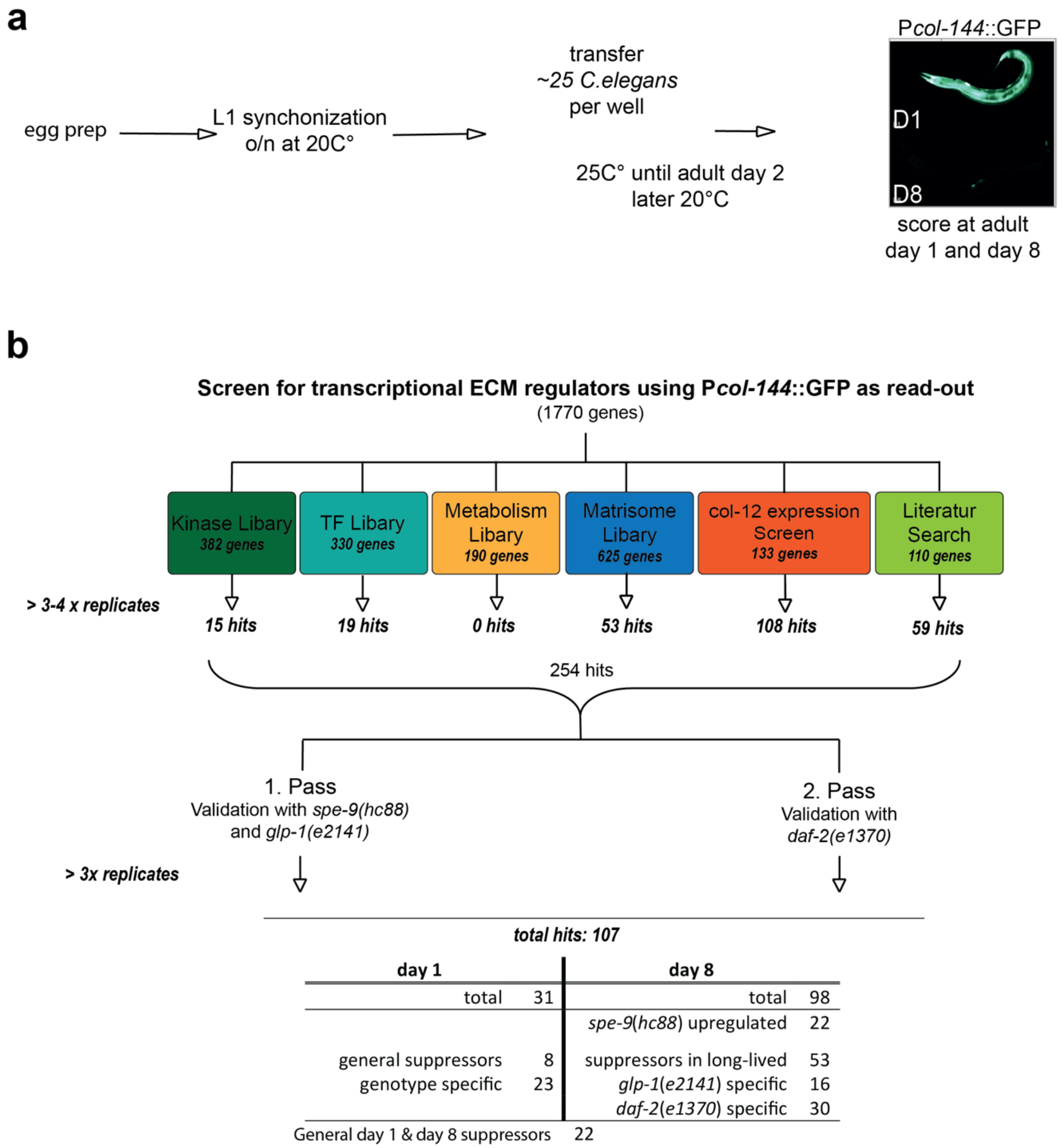

c

Category	Number	Percentage
Matrisome	49	45.8%
Signalling	12	11.2%
Autophagy	9	8.4%
Adhesome	6	5.6%
Metabolism	6	5.6%
Transcription factor	6	5.6%
Proteolysis	3	2.8%
Stress response	3	2.8%

Category	Number	Percentage
Unknown	3	2.8%
Development	2	1.9%
Muscle function	2	1.9%
Nucleic acid binding	2	1.9%
Chaperone	1	0.9%
Cytoskeleton	1	0.9%
Neuronal function	1	0.9%
Protein modification	1	0.9%

Supplementary Fig. 7. RNAi screen setup and results
(a) Schematic workflow of the screen.

(b) Schematic overview of the targeted RNAi screen. 1770 RNAi clones were screened in a first pass using transgenic *C. elegans* strains expressing the P*col-144*::GFP reporter in either a *spe-9*(*hc88*) or a long-lived *glp-1*(*e2141*) background. These hits were re-validated in *spe-9*(*hc88*) and *glp-1*(*e2141*) and additionally in *daf-2*(*e1370*) animals identifying 107 high confident hits.
(c) Table of confident hits of RNAi screen for transcriptional ECM regulators sorted by gene ontology categories, number of hits per category, and the percentage of each category being represented in relation to all screen hits. For the categorization, the WormCat online tool was used.
For details, see Supplementary Table 10.

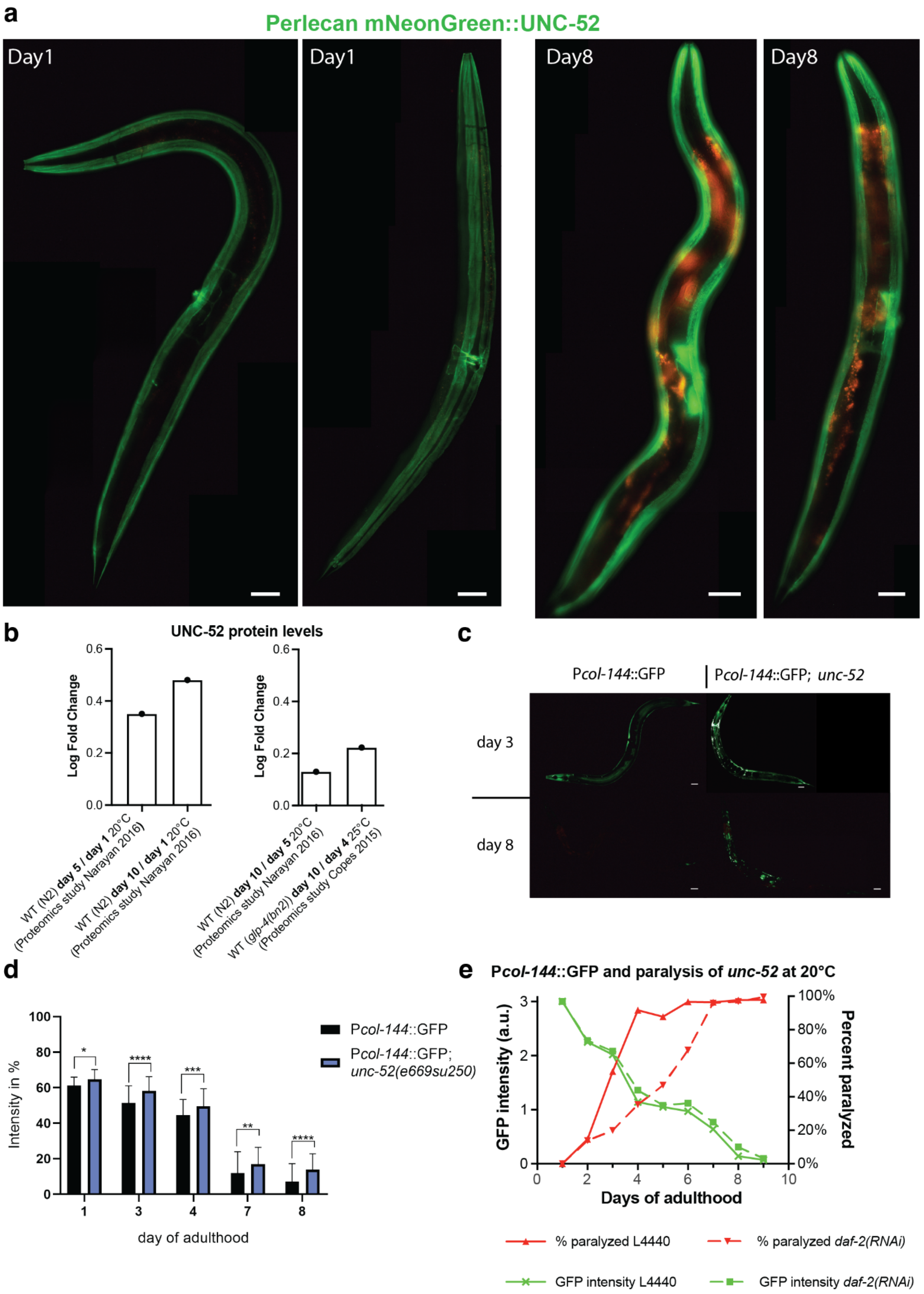
a
Perlecan mNeonGreen::UNC-52
Day1
Day1
Day8
Day8
b
UNC-52 protein levels
Log Fold Change
0.0
0.2
0.4
0.6
WT (N2) day 5 / day 1 20°C (Proteomics study Narayan 2016)
WT (N2) day 10 / day 1 20°C (Proteomics study Narayan 2016)
WT (N2) day 10 / day 5 20°C (Proteomics study Narayan 2016)
WT (glp-4(bn2)) day 10 / day 4 25°C (Proteomics study Copes 2015)
c
Pcol-144::GFP
Pcol-144::GFP; unc-52
day 3
day 8
d
Intensity in %
0
20
40
60
80
100
*
****
***
**
****
1
3
4
7
8
day of adulthood
Pcol-144::GFP
Pcol-144::GFP; unc-52(e669su250)
e
Pcol-144::GFP and paralysis of unc-52 at 20°C
GFP intensity (a.u.)
Percent paralyzed
0
1
2
3
0%
20%
40%
60%
80%
100%
0
2
4
6
8
10
Days of adulthood
% paralyzed L4440
% paralyzed daf-2(RNAi)
GFP intensity L4440
GFP intensity daf-2(RNAi)

Supplementary Fig. 8. Perlecan levels during aging and functional consequences of loss of perlecan integrity

(a) Fluorescent microscopy images of CRISPR inserted NeonGreen tagging perlecan (NK2583 *unc-52*(*qy80* [NeonGreen::UNC-52]) at days 1 and 8 of adulthood. Scale bar = 50 µm.

(b) Relative increase of UNC-52 perlecan levels during aging quantified from two different proteomics studies (See Supplementary Table 4 for details).

(c) Fluorescent images of P*col-144*::GFP; *unc-52(e669su250)* mutants at day 3 and 8 of adulthood at 20°C compared with P*col-144*::GFP in a wild-type background. Scale bar = 50 µm.

(d) Quantification of P*col-144*::GFP fluorescence in *unc-52(e669su250)* temperature-sensitive mutants at semi-permissive 20°C during aging. Three rounds, each n=20. See Supplementary Table 12 for statistics and details.

(e) Longevity intervention *daf-2(RNAi)* postponed the perlecan *unc-52(e669, su250)* mutant paralysis phenotype (dashed red line) but failed to prolong collagen expression (P*col-144*::GFP, green dashed line) during aging at semi-permissive temperature 20°C. For details, see Supplementary Table 12.

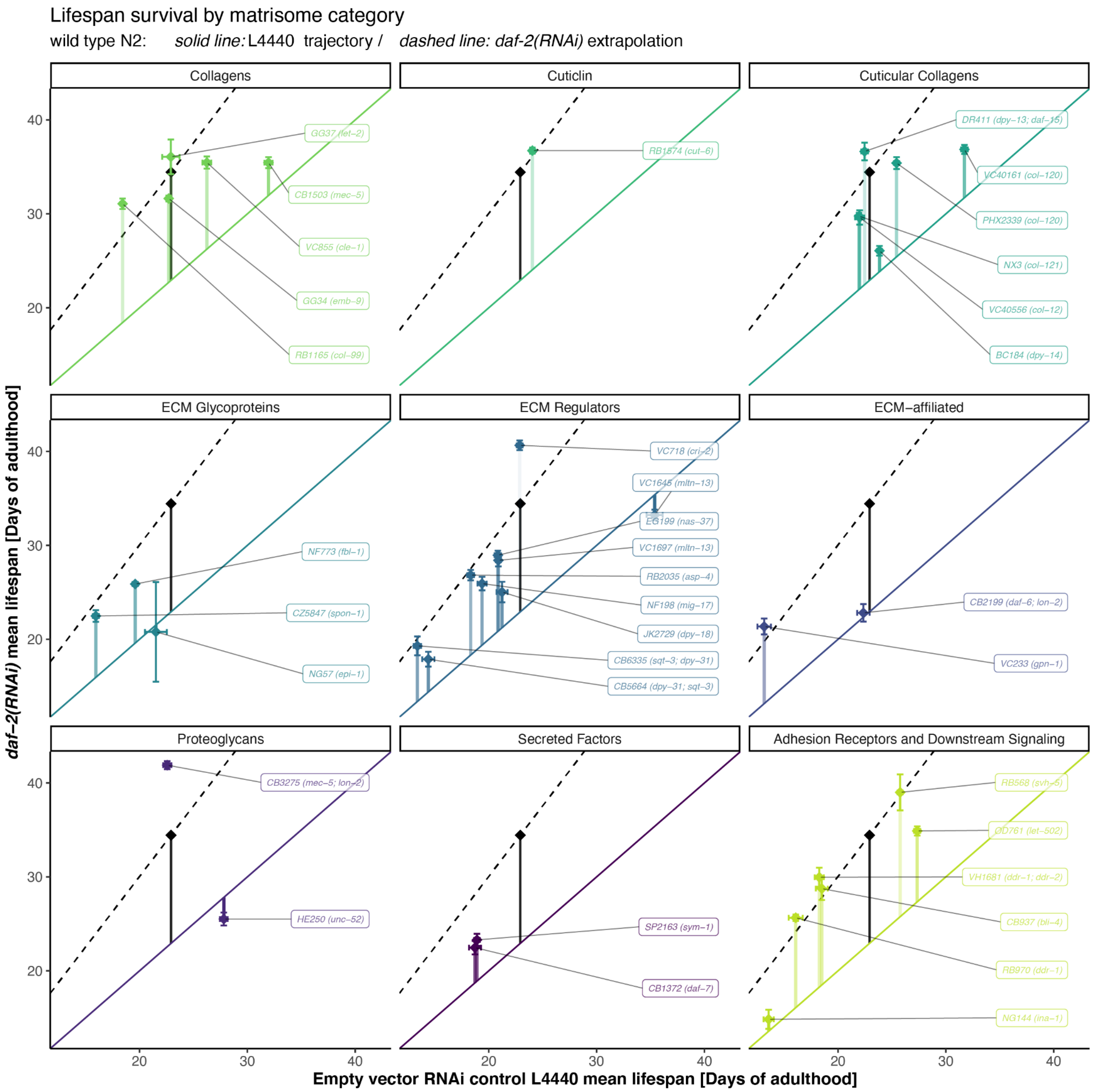

Supplementary Fig. 9. All matrisome categories are implicated in promoting healthy aging

Lifespan differences between EV (x-axis) and *daf-2 (y-axis)* RNAi treatment are displayed for all matrisome mutants grouped by matrisome category. The solid diagonal (y = x) represents the 'no effect line', and the dashed line represents the lifespan ratio of the overall global wild type across all runs comparing *daf-2* vs. EV RNAi. Each genotype is labeled in its corresponding facet and color while the wild type is shown in black. All details, raw data, and statistics are in Supplementary Table 7.

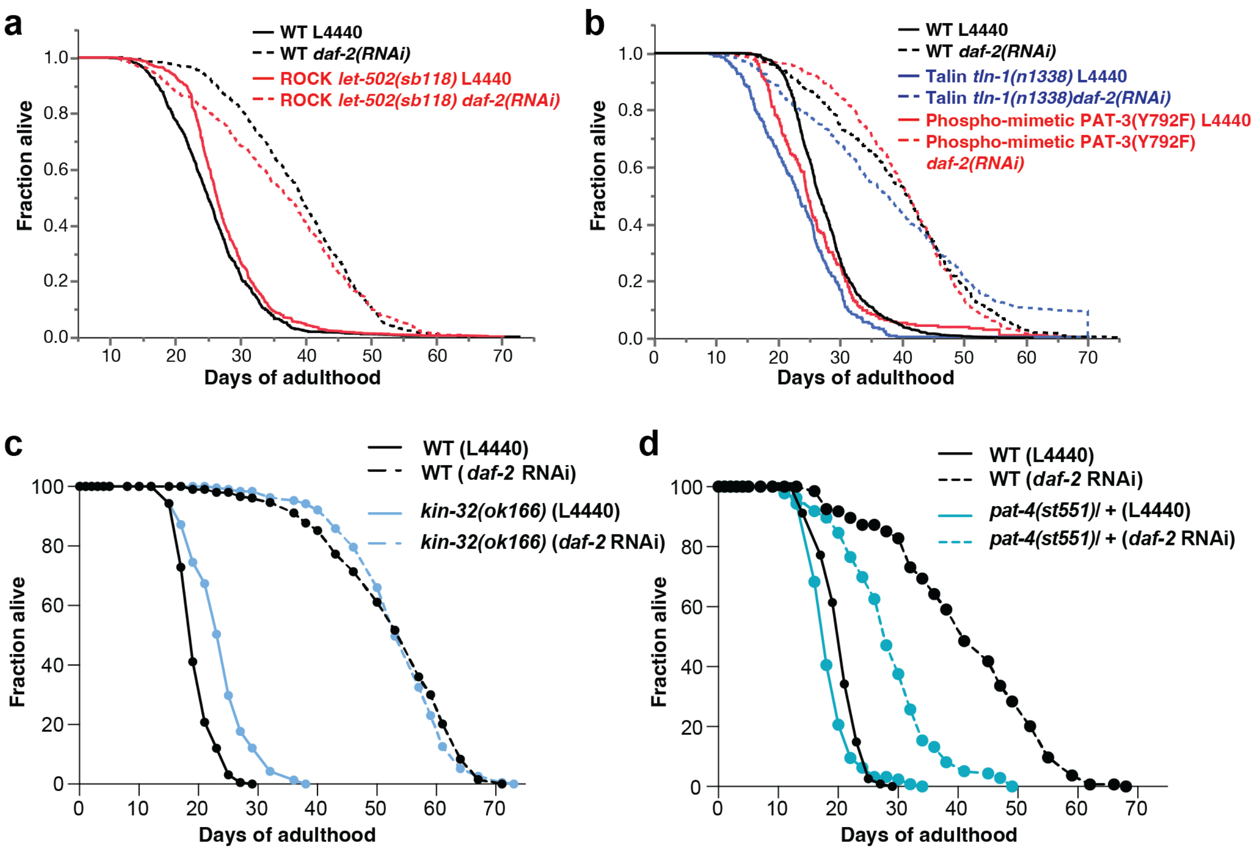

Supplementary Fig. 10. Mechanotransductive signaling genes and their functional role in longevity

(a) The integrin downstream ROCK kinase was not required for longevity at 20°C.

(b) Both the phospho-mimetic PAT-3(Y792F) that activates talin signaling and *tln-1(n1338)* mutants were not required for longevity at 20°C.

(c) Mutation in focal adhesion kinase *kin-32(ok166)* increased lifespan compared to wild type but did not suppress *daf-2(RNAi)* mediated longevity at 20°C.

(d) Heterozygous lethal mutation in *pat-4(st551)*/+ shortened WT lifespan and longevity upon reduced Insulin/IGF-1 receptor signaling at 20°C.

(a-d) For a more detailed results description, raw data, and statistical details, see Supplementary Table 7.

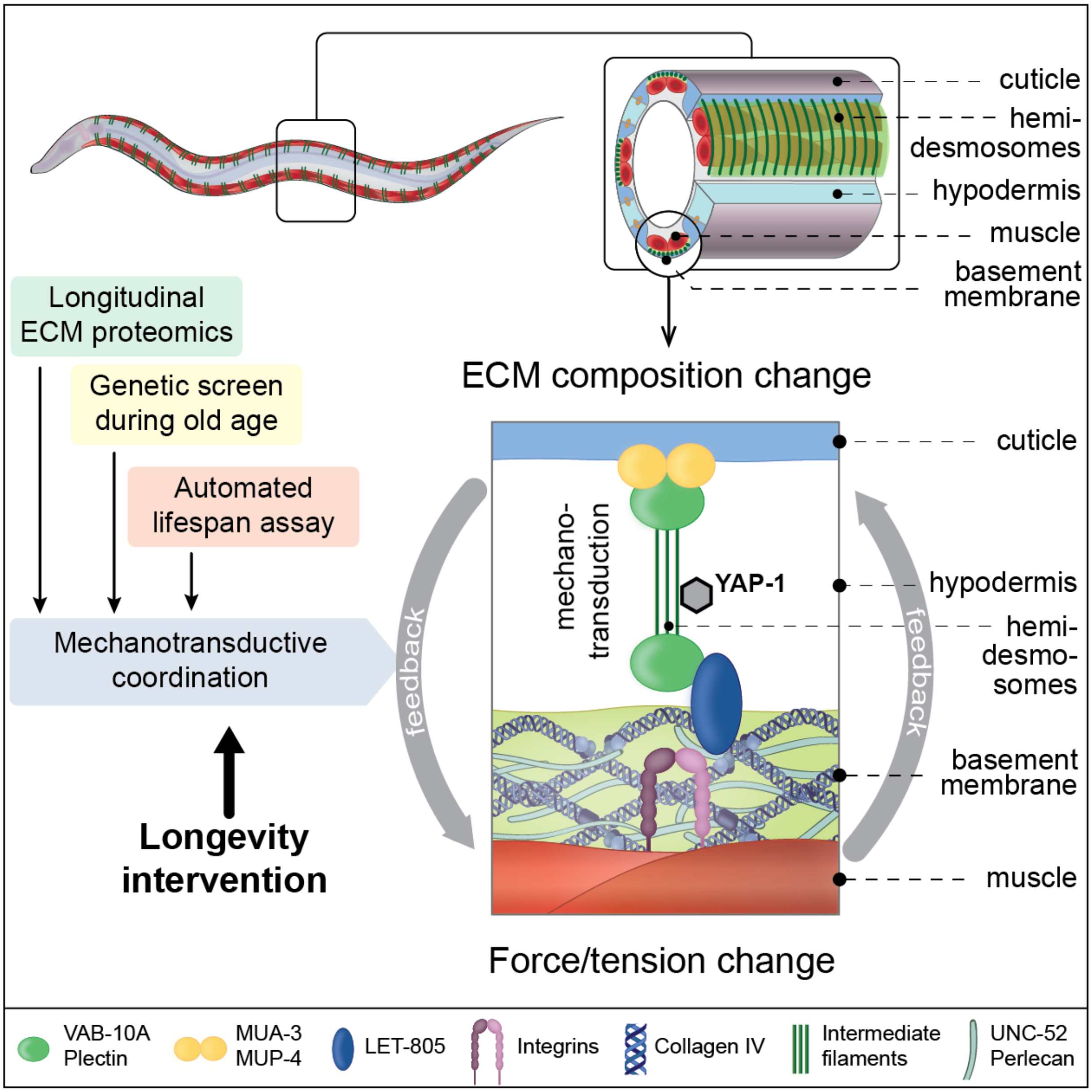

Supplementary Fig. 11. Graphical model.

SUPPLEMENTARY DISCUSSION

Proposed biomechanical model of ECM homeostasis and longevity

Based on our findings here, we propose a model that is adapted from the mechanobiological regulation of arterial walls, which includes the interactions of smooth muscles, endothelial cells, fibroblasts, and ECM remodeling (by Humphrey and Schwartz 2021) [1]. The mechanical homeostasis model requires an input (mechanical stimulus), a set point to where the homeostasis should be regulated back (negative feedback), a sensor telling the actual state, and an output (biological response). The cells most likely "perceive" the difference between the set points to what is sensed by the sensor and adapt to the difference by resetting the set point (homeostasis). For instance, increased blood pressure will thicken, whereas decreased blood pressure will thin the arterial wall to reset the intramural stress toward the set point. Thus, re-establishing equilibrium and stability (*i.e.,* homeostasis) by integrating mechanical stimuli with biological feedback processes.

In our proposed model (Fig. 7o), the hemidesmosomes act as the mechanical sensor and set point, integrating the mechanical stimuli coming from muscle contraction, internal (hypodermal) and external pressures, and shear forces. *C. elegans* is basically a liquid-filled tube that needs to maintain internal pressure. The hemidesmosomes (ceHD) are central since they connect through the basement membrane via the integrins to the body wall muscles and span through the hypodermis via the intermediate filaments and anchoring to the exoskeleton (the cuticle). We refer here to this as the apparatus: coupling muscle strength through the ceHD to the resistance of the cuticle). When the body wall muscles contract, the force generated needs to be transmitted via ceHD to the exoskeleton (cuticle) in order for the worm to move (like a muscle to bone to generate movement). Thus, the ceHDs couple two distant and distinct ECMs (basement membrane and cuticle) and are essential to transmit the forces from the muscle to the exoskeleton for the motility and movement of *C. elegans*.

Description of the proposed biomechanical model

Let us assume that post-development, the hemidesmosomes have established the set point and sensor, and the system is in homeostasis. When we place worms under external pressure and when the muscle pulls on the ceHDs, the deviation-actuation by the set point and sensor will be different and will lead to adapting the cuticle resistance by inducing the expression of cuticular collagens (col-120 or col-144, Fig. 7j-m). Our genetic data suggest that this mechanical deviation-actuation is translated into an upregulated gene expression of these cuticular collagens by nuclear translocation of the transcription factor YAP1. This ECM production/assembly/remodeling will subsequently strengthen the cuticle. With a certain time delay, the strengthening of the cuticle is then sensed by the pulling forces of the ceHDs, and we assume that this ultimately leads to a negative feedback loop stopping cuticular collagen expression, thereby driving the system back to equilibrium and homeostasis again.

Evidence for our proposed biomechanical model for this work and other groups

Our RNAi screen identified several molecular targets interfering with this feedback loop. Anatomically, genes that blocked this feedback loop were either components of the hemidesmosomes, responsible for building or maintaining the ceHDs or closely associated with the ceHD (Fig. 4b). Genetic findings from other groups make sense if interpreted with our ceHD biomechanical model. For instance, in our recent review [2], these genetic findings are discussed in light of a mechanobiological model of ceHDs. The paragraphs below in "..." are an adapted version of an excerpt of this review.

"The cuticular extracellular matrix (ECM) binds to specific transmembrane receptors, namely MUA-3 (referred to as fibrillin in mammals) and MUP-4 (also known as matrilin). These receptors connect to VAB-10 (plectin) located in the plasma membrane of the apical hypodermis (see Fig. 4). Intermediate filaments traverse the hypodermis and extend to the basal site, where VAB-10 once again binds to the LET-805 receptor (myotactin) for anchoring into the basement membrane ECM (see Fig. 4). The basement membrane, composed of collagen IV and UNC-52 (known as perlecan), interacts with a heterodimer formed by PAT-2 (integrin α) and PAT-3 (integrin β) on the muscle cell surface (see Fig. 4) [3]. Collectively, these elements constitute the force-bearing HD-like structure in *C. elegans* (ceHD).

In addition to their role in force transmission and cytoskeleton adaptation, the ECM proteins near ceHDs also influence ECM remodeling. For example, ECM proteins containing the zona pellucida domain, NOAH-1 and NOAH-2 (Fig. 4, screen hits), play a crucial role in maintaining mechanoreceptor potentials and cuticular ECM remodeling [4,5]. During the development of *C. elegans*, cuticular ECM remodeling takes place during molting. Interestingly, the loss of *unc-52, pat-3*, or *unc-95* (corresponding to paxillin in mammals) leads to molting defects associated with the ECM [5,6]. By contrast, mutations in muscle myosin *unc-54*, which is essential for muscle contraction, or in *unc-13*, which is important for neurotransmitter release, do not result in the expression of cuticular collagen [7]. The *C. elegans* cuticle surface is characterized by circumferential ridges known as annuli, and these annuli attach to ceHDs at the lower points called furrows [8]). Observations using scanning electron microscopy indicate abnormal, branched, or flat cuticular annuli in *unc-52* mutants, whereas *unc-13* mutants, which exhibit defects in neurotransmitter release, do not display such abnormalities [7]. This suggests that defects in unc-52/perlecan can impair the proper morphology of the cuticle ECM, as this basement membrane heparan sulfate proteoglycan is a critical component for ceHD function. Additionally, *pxn-2* (peroxidasin) is involved in promoting sulfilimine crosslinks of basement membrane collagen IV, thereby regulating its mechanical properties. Defects in *pxn-2* can be mitigated by mutations in ceHD components like *let-805, vab-10*, and *unc-52* [9], suggesting that changes in either the cuticular or basement membrane ECM are detected and mediated by ceHDs.

Aligning with the concept that ceHDs coordinate ECM remodeling through mechanical induction of gene expression, it is essential to understand the physiological effects of mechanical manipulation on the worm. Mechanical compression of ceHDs by subjecting *C. elegans* to hypergravity hampers the migration of motor neurons over the muscle, and mutations in *vab-10, unc-52*, and other ceHD components rescue neuronal migration [10]. Conversely, stretching ceHDs exposes the SH3 domain of VAB-10, enabling mechanosensitive signaling crucial for embryonic elongation [11]. Notably, hydrostatic

pressure increases the production of cuticular collagen *col-107* mRNA, along with lifespan [12]). These findings indicate that mechanical compression affects a wide range of physiological implications from development to aging."

Implications of mutations in the hemidesmosomes based on our proposed biomechanical model:

The mutation in perlecan *unc-52(e699,su250)* is from birth, meaning that these animals develop with "less intact" or weaker hemidesmosome structures when forces pull on them. The forces of the muscle will be transduced through the hemidesmosome to the cuticle, which acts as an exoskeleton and resistance for the worms to move (Graphical Abstract). Given the idea that the hemidesmosomes act as a set point and sensor (Fig. 7o), a "weaker" hemidesmosome will lead to a higher set point of cuticular collagen col-144, presumably adapting muscle-pulling force to strengthen parts of the cuticle. Presumably, muscle strength, transducer, and exoskeleton are adjusted and coupled to each other. We will refer to this coupling as an apparatus (muscle-hemidesmosome-cuticle). When we place worms under pressure, the muscles have to work harder to move and thus adapt the whole apparatus, including some cuticular collagens, which we observe as an increase of P*col-144*::GFP.

We think the hemidesmosomes are integrators and also the sensor of this force transduction. Since at normal conditions, *unc-52(e699,su250)*; P*col-144*::GFP worms have higher levels of *col-144* compared to wild type, but under pressure where wild-type P*col-144*::GFP animals show an increase in *col-144* expression, the *unc-52(e699,su250)*; P*col-144*::GFP worms do not (Fig. 7m), we think that *unc-52(e699,su250)* mutants have a higher set point from birth and thus higher P*col-144*::GFP levels but fail to adapt to the pressure since the sensor is broken.

The actuation-mediated deviation of the set point and the sensor are the readouts, and our data show that the nuclear translocation of *yap-1* is the response to this deviation, initiating the response that ultimately restores homeostasis. However, *unc-52(e699,su250)* mutants probably failed to integrate the difference between the set point and sensor, which explains that under pressure, *unc-52(e699,su250)* did not increase *col-144* gene expression (Fig. 7m). Thus, the higher P*col-144*::GFP levels of *unc-52(e699,su250)* compared to wild-type background might not be perceived as an "overexpression" in an *unc-52(e699,su250)*, but we think this is due to just a higher setpoint of *col-144* expression.

At permissive temperature (15°C), the penetrance of *unc-52(e699,su250)* mutation becomes visible during aging, as the midbody region of these mutants becomes progressively paralyzed [13]. This is due to the collapse of proteostasis in early *C. elegans* adulthood, leading to faulty proteins, such as the mutated perlecan *unc-52(e699,su250)* [13]. As the perlecan is located and couples the ceHD to the integrin, a faulty perlecan will lead to the uncoupling of the apparatus. We observed this with the *unc-52(e699,su250)* mutants appearing less defective on day 1 (Fig. 5a-b) compared to day 8, which correlates with midbody regions being mainly affected (Fig. 5b) by the age-related and progressive

functional loss observed during aging. This suggests that during aging, the progressive decline of ceHD function will abolish the biological output. Longevity interventions (reducing Insulin/IGF-1 signaling and *glp-1*) can delay the age-related decay of ceHD, presumably by improving protein homeostasis, but only to a certain extent (by 2-3 days).

Connection between integrin integrity, structure, physiology, and lifespan

Integrins transmit mechanical forces from the ECM to the cytoskeleton, and vice versa, whereby they get physically connected to the cytoskeleton by linker proteins, including talin/TLN-1 and integrin-linked kinase (ILK/PAT-4), and RHO-associated kinase (ROCK/LET-502) regulate the dynamic reorganization of cytoskeletal proteins by regulating the phosphorylation of myosin motors [14]. Adult-specific upshift to 20°C of temperature-sensitive loss-of-function ROCK/let-502 mutants did not suppress *daf-2(RNAi)* longevity (Supplementary Fig. 10a, Supplementary Table 7). While the dephosphorylation of the integrin receptors at tyrosine 792 in the membrane-proximal NPXY motif promotes β-integrin activations via talin recruitment [15], phospho-deficient integrin PAT-3(Y792F) mutants, as well as talin/tln-1 mutants treated with *daf-2(RNAi)*, were still long-lived (Supplementary Fig. 10b, Supplementary Table 7). Loss of focal adhesion kinase *kin-32* increased lifespan (Supplementary Fig. 10c, Supplementary Table 7). Heterozygous ILK/pat-4 mutants were shorter-lived and blocked *daf-2*-longevity (Supplementary Fig. 10d, Supplementary Table 7), consistent with severe *pat-4* knockdown leads to detachment of the cytoskeleton and shortening of lifespan, whereas mild *pat-4* knockdown has a mild effect on cytoskeleton detachment and increases lifespan [16]. Consistent with our screening hits and proteomics data are cytoskeleton remodelers implicated in longevity by our and other groups (Fig. 6f) [17–20]. This points towards a hemidesmosome-to-integrin-to-cytoskeleton remodeling axis to mediate downstream mechanotransduction and organismal longevity.

Implications of collagen overexpression based on our proposed biomechanical model:

Given this line of argument, then why would a multi-copy transgenic overexpression or CRISPR-cas9d activation of COL-120 [21] increase lifespan? In the case of multi-copy transgenic, thousands of *col-120* genes are present and accessible to transcription factors, but without a signal that leads to gene activation, nothing will happen. Only when there is an activation, then the signal is many-fold stronger, leading to an overproduction of collagen COL-120.

An overexpression should lead to longevity if mechanisms independent of hemidesmosome are at work. However, our genetic evidence indicates that, for some reason, we do not yet fully understand the sensor (*i.e.,* ceHD) or why the mechanical signal is required for the biological output, in this case, longevity. This idea of a mechanotransductive-licensing signal is in line with findings of oligodendrocyte progenitor cells where the mechanical signal overrides physiological signals to maintain OPC activity during aging [22].

Experimental evidence supporting improvements in healthspan with lifespan induced by our proposed collagen homeostasis mechanism

Previous studies, including from our lab, explored two distinct aspects of healthspan regulation: one involving the attenuation of age-related deterioration through the slowing aging by reduced IIS (rIIS) pathway and the other involving the overexpression of certain collagens.

Firstly, with respect to the rIIS pathway, previous findings provide evidence that our proposed mechanism plays a crucial licensing or assurance role in enhancing healthspan. The age-associated decline in pharyngeal pumping rates, an indication marker of healthspan, is significantly mitigated in *daf-2(e1370)* mutants, which exhibit reduced IIS and longevity [23]. However, at older ages (*e.g.,* on day 10 of adulthood), this pharyngeal pumping improvement was entirely abolished upon adult-specific knockdown of *col-120* [24], underscoring the indispensability of our proposed mechanism for realizing healthspan benefits. Additionally, higher lipofuscin levels, another aging-associated marker, were effectively curbed in *daf-2(e1370)* mutants but were elevated by *col-120RNAi* at day 10 of adulthood [24]. Moreover, oxidative stress resilience, a critical determinant of healthspan, was notably increased in *daf-2(e1370)* mutants, yet this benefit was nullified by adult-specific *col-120RNAi*, highlighting the specificity of our proposed mechanism [24]. Importantly, control experiments involving body length, cuticle leakage, and vulva integrity confirmed that the observed effects were specific to *col-120RNAi*, *col-10RNAi*, and *col-13RNAi* [24]. Lastly, cuticle stiffness increases with age shown by Coleen Murphy's lab [25]. This stiffness increase is slowed by *daf-2(e1370)* mutants but completely abolished by *col-120RNAi* [25], further substantiating the role of our proposed mechanism in rIIS-mediated healthspan improvements.

Conversely, the impact of collagens on healthspan by examining the role of collagen overexpression. Previous results from the Ouyang lab demonstrated that the overexpression of collagens, specifically COL-43 and COL-80, extended lifespan and enhanced resistance to oxidative stress induced by paraquat without affecting cuticle permeability [26]. This implicates collagens enhancement as a critical determinant of healthspan. Notably, we explored the post-developmental enhancement of collagen expression using dCas9-engineered *C. elegans,* revealing that inducing the expression of collagen genes, such as *col-120* and *col-10*, post-developmentally significantly increased lifespan and improved resilience to heat stress [21]. Upon dCas9-activated *col-120* expression, the small heat shock protein *hsp-16.2* expression was increased [21]. This newfound approach validates that the augmentation of *col-120* expression enhances longevity and healthspan parameters, including thermotolerance. These findings provide valuable insights into the experimental evidence supporting healthspan improvements and emphasize the importance of these mechanisms in modulating healthspan and longevity.

In summary, our data suggest a biomechanical model linking ceHD with two distant ECMs to regulate physiological outcomes important for promoting longevity.

SUPPLEMENTARY REFERENCES

1. Humphrey, J. D. & Schwartz, M. A. Vascular Mechanobiology: Homeostasis, Adaptation, and Disease. *Annu. Rev. Biomed. Eng.* **23**, 1–27 (2021).
2. Ewald, C. Y. & Nyström, A. Mechanotransduction through hemidesmosomes during aging and longevity. *J. Cell Sci.* **136**, (2023).
3. Zhang, H. & Labouesse, M. The making of hemidesmosome structures *in vivo*. *Dev. Dyn.* **239**, 1465–1476 (2010).
4. Vuong-Brender, T. T. K., Suman, S. K. & Labouesse, M. The apical ECM preserves embryonic integrity and distributes mechanical stress during morphogenesis. *Development* **144**, 4336–4349 (2017).
5. Frand, A. R., Russel, S. & Ruvkun, G. Functional Genomic Analysis of *C. elegans* Molting. *Plos Biol* **3**, e312 (2005).
6. Zaidel-Bar, R., Miller, S., Kaminsky, R. & Broday, L. Molting-specific downregulation of *C. elegans* body-wall muscle attachment sites: the role of RNF-5 E3 ligase. *Biochemical and Biophysical Research Communications* 1–6 (2010) doi:10.1016/j.bbrc.2010.04.049.
7. Broday, L., Hauser, C. A., Kolotuev, I. & Ronai, Z. Muscle–epidermis interactions affect exoskeleton patterning in *Caenorhabditis elegans*. *Dev Dynam* **236**, 3129–3136 (2007).
8. McMahon, L., Muriel, J. M., Roberts, B., Quinn, M. & Johnstone, I. L. Two Sets of Interacting Collagens Form Functionally Distinct Substructures within a *Caenorhabditis elegans* Extracellular Matrix. *Mol. Biol. Cell* **14**, 1366–1378 (2003).
9. Gotenstein, J. R., Koo, C. C., Ho, T. W. & Chisholm, A. D. Genetic Suppression of Basement Membrane Defects in *Caenorhabditis elegans* by Gain of Function in Extracellular Matrix and Cell-Matrix Attachment Genes. *Genetics* **208**, 1499–1512 (2018).
10. Kalichamy, S. S. *et al.* Muscle and epidermal contributions of the structural protein β-spectrin promote hypergravity-induced motor neuron axon defects in *C. elegans*. *Sci Rep-uk* **10**, 21214 (2020).
11. Suman, S. K. et al. The plakin domain of *C. elegans* VAB-10/plectin acts as a hub in a mechanotransduction pathway to promote morphogenesis. *Development* **146**, dev183780 (2019).
12. Watanabe, N. et al. Increased hydrostatic pressure induces nuclear translocation of DAF-16/FOXO in C. elegans. *Biochem Bioph Res Co* **523**, 853–858 (2020).
13. Ben-Zvi, A., Miller, E. A. & Morimoto, R. I. Collapse of proteostasis represents an early molecular event in *Caenorhabditis elegans* aging. *Proceedings of the National Academy of Sciences* **106**, 14914–14919 (2009).
14. Humphrey, J. D., Dufresne, E. R. & Schwartz, M. A. Mechanotransduction and extracellular matrix homeostasis. *Nature Reviews Molecular Cell Biology* **15**, 802–812 (2014).
15. Walser, M., Umbricht, C. A., Fröhli, E., Nanni, P. & Hajnal, A. β-Integrin dephosphorylation by the Density-Enhanced Phosphatase DEP-1 attenuates EGFR signaling in *C. elegans*. *PLoS genetics* **13**, e1006592 (2017).
16. Nishimura, M. et al. A dual role for integrin-linked kinase and β1-integrin in modulating cardiac aging. *Aging cell* **13**, 431–440 (2014).

17. Mansfeld, J. et al. Branched-chain amino acid catabolism is a conserved regulator of physiological ageing. *Nat Commun* **6**, 10043 (2015).
18. Baird, N. A. et al. HSF-1-mediated cytoskeletal integrity determines thermotolerance and life span. *Sci New York N Y* **346**, 360–3 (2014).
19. Vitiello, D., Dakhovnik, A., Statzer, C. & Ewald, C. Y. Lifespan-Associated Gene Expression Signatures of Recombinant BXD Mice Implicates Coro7 and Set in Longevity. *Frontiers Genetics* **12**, 694033 (2021).
20. Ewald, C. Y. et al. NADPH oxidase-mediated redox signaling promotes oxidative stress resistance and longevity through *memo-1* in *C. elegans*. *eLife* **6**, 819 (2017).
21. Goyala, A. & Ewald, C. Y. CRISPR-activated expression of collagen *col-120* increases lifespan and heat tolerance. *microPublication Biol.* **2023**, 10.17912/micropub.biology.000730 (2023).
22. Segel, M. et al. Niche stiffness underlies the ageing of central nervous system progenitor cells. *Nature* **573**, 130–134 (2019).
23. Ewald, C. Y., Castillo-Quan, J. I. & Blackwell, T. K. Untangling Longevity, Dauer, and Healthspan in *Caenorhabditis elegans* Insulin/IGF-1-Signalling. *Gerontology* **64**, 96–104 (2018).
24. Ewald, C. Y., Landis, J. N., Abate, J. P., Murphy, C. T. & Blackwell, T. K. Dauer-independent insulin/IGF-1-signalling implicates collagen remodelling in longevity. *Nature* **519**, 97–101 (2015).
25. Rahimi, M., Sohrabi, S. & Murphy, C. T. Novel elasticity measurements reveal *C. elegans* cuticle stiffens with age and in a long-lived mutant. *Biophys J* (2022) doi:10.1016/j.bpj.2022.01.013.
26. Deng, G., Li, L. & Ouyang, Y. Modeling paraquat-induced lung fibrosis in *C. elegans* reveals KRIT1 as a key regulator of collagen gene transcription. *Aging (Albany NY)* **13**, 4452–4467 (2021).
