# Supplementary material for: Longevity interventions modulate mechanotransduction and extracellular matrix homeostasis in C. elegans
Source: Nat Commun. 2024 Jan 4;15:276. doi: 10.1038/s41467-023-44409-2 (PMC10766642; doi:10.1038/s41467-023-44409-2)
Supplement: Supplementary file 3 — Description of Additional Supplementary Files [file 41467_2023_44409_MOESM3_ESM.pdf]

Description of Additional Supplementary Files

File name: Supplementary Data 1

Description: Matrisome Atlas

File name: Supplementary Data 2

Description: Aging Transcriptomics

File name: Supplementary Data 3

Description: Aging Proteomics

File name: Supplementary Data 4

Description: Collagen Levels

File name: Supplementary Data 5

Description: COL-120::Dendra

File name: Supplementary Data 6

Description: COL-19FRET

File name: Supplementary Data 7

Description: Lifespan Data

File name: Supplementary Data 8

Description: EMB-9::Dendra

File name: Supplementary Data 9

Description: ECM plasticity and longevity proteomics

File name: Supplementary Data 10

Description: RNAi screen hits

File name: Supplementary Data 11

Description: Collagen EMB-9::mCherry with integrin PAT-3::GFP colocalization data.

File name: Supplementary Data 12

Description: Reporter col-144 promoter driven GFP data

File name: Supplementary Data 13

Description: YAP-1::GFP

File name: Supplementary Data 14

Description: Pressure induced collagen promoter driven GFP data.
